# Restrictive intraoperative fluid management was associated with higher incidence of composite complications compared to less restrictive strategies in open thoracotomy: A retrospective cohort study

**DOI:** 10.1038/s41598-020-65532-w

**Published:** 2020-05-21

**Authors:** Jie Ae Kim, Hyun Joo Ahn, Ah Ran Oh, Jisun Choi

**Affiliations:** Department of Anesthesiology and Pain Medicine, Samsung Medical Center, Sungkyunkwan University School of Medicine, 81 Irwon-ro Gangnam-gu, Seoul, 06351 South Korea

**Keywords:** Oncology, Risk factors

## Abstract

Restrictive fluid management has been recommended for thoracic surgery. However, specific guidelines are lacking, and there is always concern regarding impairment of renal perfusion with a restrictive policy. The objective of this study was to find the net intraoperative fluid infusion rate which shows the lowest incidence of composite complications (either pulmonary complications or acute kidney injury) in open thoracotomy. We hypothesized that a certain range of infusion rate would decrease the composite complications within postoperative 30 days. All patients (n = 1,031) who underwent open thoracotomy at a tertiary care university hospital were included in this retrospective study. The time frame of fluid monitoring was from the start of operation to postoperative 24 hours. The cutoff value of the intraoperative net fluid amount was 4–5 ml.kg^−1^.h^−1^ according to the minimum p-value method, thus, patients were divided into Low (≤3 ml.kg^−1^.h^−1^), Cutoff (4–5 ml.kg^−1^.h^−1^) and High (≥6 ml.kg^−1^.h^−1^) groups. The Cutoff group showed the lowest composite complication rate (19%, 12%, and 13% in the Low, Cutoff, and High groups, respectively, P = 0.0283; Low vs. Cutoff, P = 0.0324, Bonferroni correction). Acute respiratory distress syndrome occurred least frequently in the Cutoff group (7%, 3%, and 6% for the Low, Cutoff, and High groups, respectively, P = 0.0467; Low vs. Cutoff, P = 0.0432, Bonferroni correction). In multivariable analysis, intraoperative net fluid infusion rate was associated with composite complications, and the Cutoff group decreased risk (odds ratio 0.54, 95% confidence interval: 0.35–0.81, P = 0.0035). In conclusion, maintaining intraoperative net fluid infusion at 4–5 ml.kg^−1^.h^−1^ was associated with better results in open thoracotomy, in terms of composite complications, compared to more restrictive fluid management.

## Introduction

Postoperative pulmonary complications are the leading cause of morbidity, mortality, and prolonged hospital stay after thoracic surgery^1^. Fluid overload is one of the major risk factors^2–4^ and is receiving significant attention as a preventable risk factor. Therefore, fluid restriction is usually recommended during and after thoracic surgery^2–6^. Despite the well-known risk of fluid overload, few studies have provided a guideline for intraoperative fluid management. Some experts insist on extreme restriction, and even zero fluid balance, for thoracic surgery^5^. However, evidence for the efficacy of zero balance only exists in the context of intensive care units (ICUs)^7^. Guidelines for ICU patients may not be applicable to patients undergoing a major operation because of anesthesia-induced vasodilation, increased insensible loss from the open thoracic cavity, surgical trauma induced fluid shift, and hemodynamic instability related to surgery. In addition to the lack of solid evidence, impairment of perfusion of major organs, especially the kidney, is another major concern with restrictive fluid management.

The recent increase in acute kidney injury (AKI) incidence is mainly due to a change to the definition of AKI and an increase in the size of the elderly population with comorbidities; nevertheless, widespread restrictive fluid management and subsequent reduction of circulatory volume during operations may also have contributed to the increase in AKI^8,9^. Recent large trial (RELIEF) concluded restrictive fluid management increases AKI compared to liberal fluid management after major abdominal surgery^9^. There has been growing interest in tissue hypoperfusion resulting from inadequate fluid resuscitation and the development of AKI after lung resection surgery too^10,11^. Data now show that the risk of AKI after lung resection surgery ranges between 6% and 33%^11–13^.

Therefore, there is an urgent need to determine the optimal maintenance fluid infusion rate which balances the risk between pulmonary and renal complications during major thoracic surgery. Previous studies focused on one organ, mostly the lungs in relation to fluid amount^2–5,14^.

The objective of the current study was to compare composite complications (pulmonary or renal complications) among patients who received fluid infusion at varying rates through retrospective analysis. It is difficult to perform large randomized controlled trials featuring a wide range of infusion rates because restrictive fluid prescribing is a widespread practice. Therefore, analyzing retrospective data could be a valuable option. We only included open lung resection, where insensible fluid loss is higher, and thus fluid management more critical, than in video-assisted thoracoscopic surgery (VATS). We hypothesized that a certain range of infusion rate would decrease the composite complications within postoperative 30 days.

## Results

There were no significant differences in demographic characteristics, underlying comorbidities, or operational characteristics among the three groups, except that operation duration was shorter and TNM stage 3 or 4 was less frequent in the High group (Table 1).

**Table 1:** Baseline characteristics of patients and operations.

Variables	Low (*n* = 439)	Cutoff (*n* = 412)	High (*n* = 180)	P
Preoperative data				
Age, yr	64 ± 9	64 ± 9	65 ± 9	0.85
Male, n (%)	350 (80)	313 (76)	140 (78)	0.42
ASA physical status ≥3, n (%)	37 (8)	25 (6)	7 (4)	0.10
Current smoker, n (%)	90 (21)	91 (23)	45 (27)	0.42
Heavy drinking, n (%)	49 (11)	35 (9)	23 (13)	0.23
TNM Stage 3, 4, n (%)	138 (31)	133 (32)	39 (22)	0.0248
Neoadjuvant therapy, n (%)	97 (22)	98 (24)	32 (18)	0.27
Comorbid condition, n (%)				
Hypertension	184 (42)	156 (38)	68 (38)	0.42
Diabetes mellitus	81 (19)	71 (17)	26 (14)	0.49
Pulmonary dysfunction	48 (11)	48 (11)	27 (15)	0.34
Cardiac disease	26 (6)	26 (6)	5 (3)	0.20
Cerebrovascular disease	13 (3)	15 (4)	9 (5)	0.46
Chronic renal failure	4 (1)	2 (0)	0 (0)	0.38
Intraoperative data				
Colloid, n (%)	181 (41)	200 (49)	84 (47)	0.09
Type of surgery, n (%)				0.70
Wedge resection	16 (4)	13 (3)	5 (3)	
Segmentectomy	11 (3)	11 (3)	6 (3)	
Lobectomy	355 (81)	347 (84)	153 (85)	
Sleeve lobectomy	57 (13)	40 (10)	16 (9)	
Pneumonectomy	0 (0)	1(0)	0 (0)	
Use of inotrope, n (%)	83 (19)	78 (19)	32 (18)	0.94
Use of vasopressor, n (%)	176 (40)	187 (45)	83 (46)	0.21
Operation duration, min	199 [161, 254]	196 [161, 242]	179 [146, 227]	0.0004
Thoracic epidural analgesia, n (%)	193 (44)	165 (40)	65 (36)	0.27

Values are presented as mean ± SD, median [interquartiles] or n (%). Abbreviations: ASA American society of anesthesiologist, CCRT concurrent chemoradiotherapy. TNM tumor node metatstasis. TNM stage and operation duration: P < 0.05 between Group High vs. Group Low & Cutoff. Bonferroni correction was done.

Input and output data are shown in Table 2. Intraoperative net fluid infusion rate was 2.4 ± 0.8 ml.kg^−1^.h^−1^, 4.4 ± 0.5 ml.kg^−1^.h^−1^, 6.9 ± 1.2 ml.kg^−1^.h^−1^ (P < 0.0001, all groups differ each other). Colloid solution was administered in 181/439 (41%), 200/412 (49%), 84/180 (47%) in the Low, Cutoff and High groups, respectively (P = 0.09, Table 1). Among colloids, hydroxyethyl starch solution was administered in 175/439 (40%), 192/412 (43%), 82/180 (46%) in the Low, Cutoff and High groups, respectively (P = 0.89). Intraoperative blood loss and urine output were higher in the Low group than the other groups (Table 2).

**Table 2:** Input and output data.

Variables	Low (n = 439)	Cutoff (*n* = 412)	High (*n* = 180)	P
**Operation**				
Fluid amount, ml	1400[1112, 1700]	1700[1400, 2025]	2000[1600, 2365]	<0.0001
Estimated blood loss, ml	250[200, 400]	200[150, 350]	200[150, 300]	0.0034
Urine output, ml	340[216, 514]	240[133, 330]	223[132, 333]	<0.0001
Total infusion rate	4.9 ± 1.4	6.4 ± 1.2	9.3 ± 1.9	<0.0001
Net infusion rate	2.4 ± 0.8	4.4 ± 0.5	6.9 ± 1.2	0.0001
Colloid infusion rate	0.8 ± 1.0	1.0 ± 1.0	1.6 ± 1.3	0.001
**ICU 24 hour**				
Fluid amount, ml	1480[1185, 1844]	1382[1061, 1667]	1349[1066, 1668]	0.0009
Chest bottle, ml	460[350, 600]	480[370, 600]	459[360, 645]	0.34
Urine output, ml	1048[830, 1325]	1078[845, 1365]	1027[845, 1330]	0.29
Net fluid balance, ml	−75[−402, 276]	−219[−569, 75]	−216[−515, 100]	0.0003
Body weight change, kg	0[−0.7, 0]	0[−0.3, 0]	0[−0.2, 0]	0.22

Values are presented as median [interquartiles] or mean ± standard deviation. Body weight change: difference between pre- and after 24 hours of operation. Intraoperative fluid amount: P < 0.05 between each group. Intraoperative estimated blood loss and urine output: P < 0.05 between Group Low vs. Group Cutoff & High. Net fluid balance: P < 0.05 between Group Low vs. Group Cutoff & High. Bonferroni correction was done.

The median body weight change was 0 kg between the preoperative period and 24 hours after operation and did not differ among the three groups. All three groups maintained negative fluid balance at ICU. However, the Low group showed higher amount of fluid administration and less negative fluid balance compared to the other groups in ICU (Table 2).

**Figure 1:**
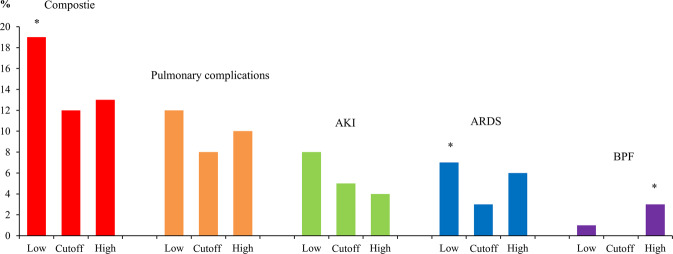
Incidence of complication between three infusion groups. *P < 0.05 compared to the other groups.

The incidence of composite complications (pulmonary complications or AKI) was lowest in the Cutoff group (19%, 12%, and 13% in Low, Cutoff, and High, respectively, P = 0.0283; Low vs. Cutoff, P = 0.0324, Bonferroni correction). Among individual complications, ARDS occurred less frequently in the Cutoff group (7%, 3%, and 6% in the Low, Cutoff, and High groups, respectively, P = 0.0467; Low vs. Cutoff, P = 0.0432, Bonferroni correction) and bronchopleural fistula occurred more commonly in the High group (1%, 0%, and 3%, respectively, P = 0.0081; Cutoff vs. High, P = 0.0339, Bonferroni correction). AKI rates were not different between the groups (8%, 5%, and 4% in the Low, Cutoff, and High groups, respectively, P = 0.16), but showed decreasing trend with increasing fluid amount (P for trend = 0.0711). Other complications and duration of ICU and hospital stays were not different among the groups (Fig. 1, Table 3).

**Table 3:** Postoperative complications.

Complication	Low (*n* = 439)	Cutoff (*n* = 412)	High (*n* = 180)	P
Composite complication	81 (19)	50 (12)	24 (13)	0.0283
Pulmonary complication	53 (12)	34 (8)	18 (10)	0.18
ARDS	30 (7)	13 (3)	11 (6)	0.0467
Pneumonia	24 (6)	22 (5)	11 (6)	0.93
Atelectasis requiring bronchoscopy	8 (2)	5 (1)	1 (1)	0.44
AKI	34 (8)	21 (5)	8 (4)	0.16
Stage 1	29	17	8	
Stage 2	3	3	0	
Stage 3	2	1	0	
Other complications				
Surgical infection total	8 (2)	8 (2)	5 (3)	0.74
Empyema	5 (1)	4 (1)	4 (2)	0.43
Incision site infection	0 (0)	2 (1)	1 (1)	0.32
Wound dehiscence	3 (1)	2 (1)	0 (0)	0.38
Bronchopleural fistula	3 (1)	1 (0)	5 (3)	0.0081
Arrhythmia (A.fib)	53 (12)	55 (13)	17 (9)	0.41
Arrhythmia (non A.fib)	23 (5)	20 (5)	7 (4)	0.77
Myocardial infarction	1 (0)	1 (0)	0 (0)	0.80
Cerebral infarction	2 (1)	2 (1)	0 (0)	0.65
Prolonged effusion	8 (2)	9 (2)	5 (3)	0.75
Prolonged air leak	36 (8)	24 (6)	20 (11)	0.08
Pulmonary thromboembolism	2 (0)	0 (0)	0 (0)	0.26
Hospital days, day	8 [6, 11]	8 [6, 10]	7 [6, 11]	0.81
ICU stay, day	1 [1, 2]	1 [1, 2]	1 [1, 1]	0.13
In-hospital Death	10 (2)	9 (2)	3 (2)	0.88

Values are presented as patient number (%) or median [interquartiles]. Abbreviations: ARDS acute respiratory distress syndrome, AKI acute kidney injury, A.fib atrial fibrillation. Composite complication: P = 0.0324 between Group Low vs. Group Cutoff. ARDS: P = 0.0432 between Group Low vs. Group Cutoff. Bronchopleural fistular: P = 0.0339 between Group Cutoff vs. Group High. Bonferroni correction was done.

Multivariable analysis showed that fluid infusion rate was independently associated with composite complications, and the Cutoff group showed a lower risk of composite complications [odds ratio (OR) 0.54, P = 0.0035]. Other risk factors for composite complications were age (per year OR 1.05, P = 0.0001), male sex (OR 2.01, P = 0.0026), ASA physical status ≥3 (OR 2.00, P = 0.0403), current smoker (OR 1.57, P = 0.0453), underlying lung disease (OR 1.98, P = 0.0065), chronic renal disease (OR 12.0, P = 0.0242), Neoadjuvant therapy (OR 1.86, P = 0.0144), duration of operation (per hour OR 1.33, P = 0.0017), use of hydroxyethyl starch (OR 1.56, P = 0.0220), and inotropes (OR 1.76, P = 0.0116) (Table 4).

**Table 4:** Multivariable analysis for composite complication.

Variables	Univariable			Multivariable		
	OR	95% CI	P	OR	95%CI	P
Group						
Low	Reference			Reference		
Cutoff	0.61	0.42, 0.89	0.0112	0.54	0.35, 0.81	0.0035
High	0.68	0.42, 1.11	0.13	0.60	0.35, 1.05	0.07
Age, per year	1.05	1.03, 1.07	<0.0001	1.05	1.02, 1.08	0.0001
Male	3.00	1.72, 5.22	0.0001	2.01	1.09, 3.71	0.0026
ASA ≥3	2.70	1.57, 4.66	0.0003	2.00	1.03, 3.88	0.0403
Diabetes mellitus	1.86	1.24, 2.78	0.0025			
Hypertension	1.27	0.90, 1.79	0.17			
Heavy drinking	1.54	0.93, 2.55	0.09			
Current smoker	1.57	1.07, 2.31	0.0210	1.57	1.01, 2.45	0.0453
Lung disease	2.27	1.45, 3.55	0.0003	1.98	1.2, 3.24	0.0065
Heart disease	1.22	0.60, 2.46	0.59			
Chronic renal disease	5.74	1.15, 28.7	0.0333	12.0	1.4, 104.7	0.0242
Cerebral disease	1.87	0.86, 4.04	0.11			
Neoadjuvant	1.18	0.79, 1.76	0.42	1.86	1.13, 3.05	0.0144
TNM Stage 3 & 4	0.94	0.65, 1.37	0.76			
Thoracic epidural	1.00	0.82, 1.23	0.99			
Operation type	1.13	0.80, 1.58	0.49			
Operation duration, per h	1.30	1.12, 1.50	0.0004	1.33	1.11, 1.59	0.0017
Hydroxyethyl starch	2.06	1.45, 2.91	<0.0001	1.56	1.07, 2.30	0.0220
Use of inotrope	2.08	1.41, 3.08	0.0002	1.76	1.14, 2.74	0.0116
Use of vasopressor	1.63	1.14, 2.32	0.0069			

All variables underwent univariable and multivariable analysis. Abbreviations: OR odds ratio, CI confidence interval.

## Discussion

In the current study, an intraoperative net fluid infusion of 4–5 ml.kg^−1^.h^−1^ showed lower rates of composite complications and ARDS than ≤3 ml.kg^−1^.h^−1^ in open thoractoomy. In multivariable analysis, intraoperative net fluid infusion rate was independently associated with composite complications, and a rate at 4–5 ml.kg^−1^.h^−1^ reduced the risk.

While avoiding too much fluid is generally agreed upon as being important^6,15,16^, the evidence for severe restriction or zero balance is not strong for intraoperative management during thoracic surgery^5^. In this study, the optimal intraoperative net fluid infusion rate which was determined by the minimum p-value method for the lowest complications was 4–5 ml.kg^−1^.h^−1^, not ≤2 ml.kg^−1^.h^−1^ which is previously recommended infusion rate in the systematic review for thoracic surgery^17^. Previous studies included both VATS and open surgery^4,17,18^, and combined both intra- and postoperative fluid amount. Mostly, net infusion rate was 1–4 ml.kg^−1^.h^−1^ in addition to the replacement of losses^4,18^. Therefore, our study has a merit in terms that we only focused on intraoperative fluid management, tested wider range of fluid infusion rates (1–11 ml.kg^−1^.h^−1^), and dealt with open thoracotomy which is quite different from VATS in fluid requirement. Our results are against restrictive fluid management strategy. Accordingly, a trend of pursuing normovolemia is now drawing attention in thoracic surgery^10^.

In our study, the difference of fluid amount between the Low and High groups was only around 600 ml (300 ml between each group). In addition, all three groups maintained negative fluid balance at ICU reflecting current fluid policy. Previous studies usually compared excessive fluid differences in ICU (for example, −136 ml vs. +6,992 ml)^19^. We found that the small difference in fluid amount was also related to a different incidence of complications after thoracic surgery. In the systematic review of thoracic surgery, the mean difference of intraoperative fluid volume was only 460 ml, and the difference of cumulative intra- and postoperative fluid infusion rate was only 0.6 ml.kg^−1^.h^−1^ between the conservative and liberal fluid groups, and this small difference was related to the development of ARDS after thoracic surgery^4,17^. Odds ratio for the development of respiratory distress syndrome was 1.42 per 1 ml.kg^−1^.h^−1^ increase in cumulative intra- and postoperative fluid amount^18^.

Possible etiology is that higher blood loss and urine output were not adequately replaced in the Low group because of strict adherence to fluid restriction policy during surgery. The Low group subsequently required higher amount of fluid and showed less negative fluid balance at ICU than other groups, probably to compensate hypovolemic state during operation. This intraoperative hypo-perfusion and delayed resuscitation may have resulted in higher complications. The endothelial glycocalyx plays a key role in endothelial barrier function and lung injury, and is destroyed by both hypovolemia (ischemia) and rapid large volume challenge (shear stress)^20,21^. Therefore, our study may indicate not only fluid amount but also timing of fluid administration is important for postoperative complications. Hemodynamic instability induced by anesthesia and operation needs to be addressed actively with adequate fluid resuscitation, instead of adhering to a restriction policy to prevent damage of endothelial glycocalyx layer. It is also notable that the ICU physicians were able to maintain more negative fluid balance in the Cutoff group than in the Low group (−75 ml, −219 ml, −216 ml, Low, Cutoff, and High group respectively), which helps practice of fluid restriction in ICU.

Another possible reason for the lack of benefit of an intraoperative restrictive policy is the mechanism of ARDS. Pulmonary edema does not easily occur due to fluid administration because of pulmonary capillary recruitment and distention. In fact, until the left atrial pressure is doubled, pulmonary capillary filtration does not increase markedly^22^. Accordingly, a fluid protocol targeting normovolemia did not increase extravascular lung water in patients undergoing elective lung resection surgery^23^. On the contrary, ARDS is by definition a syndrome of inflammation and increased permeability. Thus, ARDS would occur regardless of fluid restriction^24^.

Goal-directed fluid therapy (GDFT) based on the variations of pulse pressure and stroke volume^25^, or based on stroke volume or cardiac output itself^26^ may be considered for thoracic surgery instead of fixed fluid infusion. However, the variations of pulse pressure and stroke volume are relatively inaccurate in thoracic surgery due to open chest, one lung ventilation, low tidal volume, etc^25^. Another shortcoming of GDFT is the possibility of fluid overload. 200 ml of fluid was challenged each time for a 10% increase of stroke volume (positive response) as a GDFT protocol in thoracic surgery^26^. This challenge technique, however, can be dangerous because positive fluid response does not necessarily mean that the patient is true hypovolemic. It can occur in various situations, such as vasodilation from anesthetics^27^ or reduction of venous return from any cause^28^. This may lead to unnecessary fluid overload, especially in thoracic surgery accompanied by no apparent blood loss.

In our study, AKI rates were 8%, 5%, and 4% in the Low, Cutoff, and High groups, respectively (P = 0.16, P for trend=0.0711). There was a decreasing tendency of AKI with increasing amount of fluid. A previous study reported that intraoperative fluid amount ≤3 ml.kg^−1^.h^−1^ did not increase the incidence of AKI (retrospective study)^29^. In addition, an intraoperative fluid amount of 2 ml.kg^−1^.h^−1^ did not decrease urine output nor increase serum creatinine level compared to 8 ml.kg^−1^.h^−1^ (randomized controlled study)^30^. Most cases were VATS in previous studies^29,30^. VATS is known to be associated with less fluid loss and hemodynamic changes than open thoracotomy^29^. Our results suggested a trend between more fluid restriction and higher AKI risk in open thoracotomy. Recent RELIEF trial (n = 3,000) also supports liberal fluid management over restrictive fluid management to reduce AKI in major abdominal surgery^9^.

In our multivariable analysis, other risk factors for composite complications were age, male sex, ASA physical status ≥3, current smoker, underlying lung disease, chronic renal disease, neoadjuvant therapy, longer duration of operation, use of hydroxyethyl starch and inotropes. Modifiable risk factor in addition to fluid management, was smoking and the use of hydroxyethyl starch and inotropes. Smoking is encouraged to stop^31^. Hydroxyethyl starch was associated with AKI in the latest meta analysis^32^ and in the previous studies for thoracic surgery^29^, therefore, better be avoided in high risk patients. Fluid restriction and use of inotrope/vasopressor are usually concurrent^17^. However, our results suggest the use of inotropes does not overcome fluid restriction, and maintaining blood pressure in the fluid restricted patient would not fix the problem of hypoperfusion^33^.

Previously, the occurrence of postoperative pulmonary complications was more frequent when the intraoperative infusion rate exceeded 6 ml.kg^−1^.h.^−16^ We did not observe higher incidences of postoperative complications with an net infusion rate of ≥6 ml.kg^−1^.h^−1^ except bronchopleural fistula. Our study was not powered for this complication and previous studies are rare on this regard. The relationship between excessive fluid infusion and bronchopleural fistula requires further studies^34,35^.

We used net infusion rate (input – output) based on previous studies^4,18^. Some studies used total infusion rate without considering output^6,16^. In our data, increase of total infusion rate was associated with the reduced risk of composite complications (total infusion rate cutoff 5–6 ml.kg^−1^.h^−1^ by minimum p-value method; composite complications 21%, 15%, 12%, Low, Cutoff, and High groups, respectively, P = 0.0239, P for trend=0.0077, data not shown). Net infusion seems to be more relevant because blood loss and urine output differ by patient. Accordingly, net infusion rate was associated with acute lung injury but total infusion rate was not, in the study of Licker *et al*.^4^.

This study had some limitations. First, it used a retrospective design, where uncontrolled data and underreporting are inherent drawbacks of such studies. Second, we only included open lung resection cases from a single center. Thus, our fluid recommendations may not be applicable to less invasive thoracic surgeries, such as VATS, or to other hospitals using different patient care protocols. Third, personal preference is playing an important role in perioperative fluid strategies in thoracic surgery, especially since there has been no strong evidence that one fluid regimen is superior to another. Our result is the reflection of current practice and could be used as a platform of future prospective studies.

In conclusion, maintaining net fluid infusion at 4–5 ml.kg^−1^.h^−1^ was associated with better results, in terms of composite complications, compared to more restrictive fluid management. Thus, optimal fluid management warrants more attention in open lung resection surgery.

## Methods

### Study population

**Figure 2:**
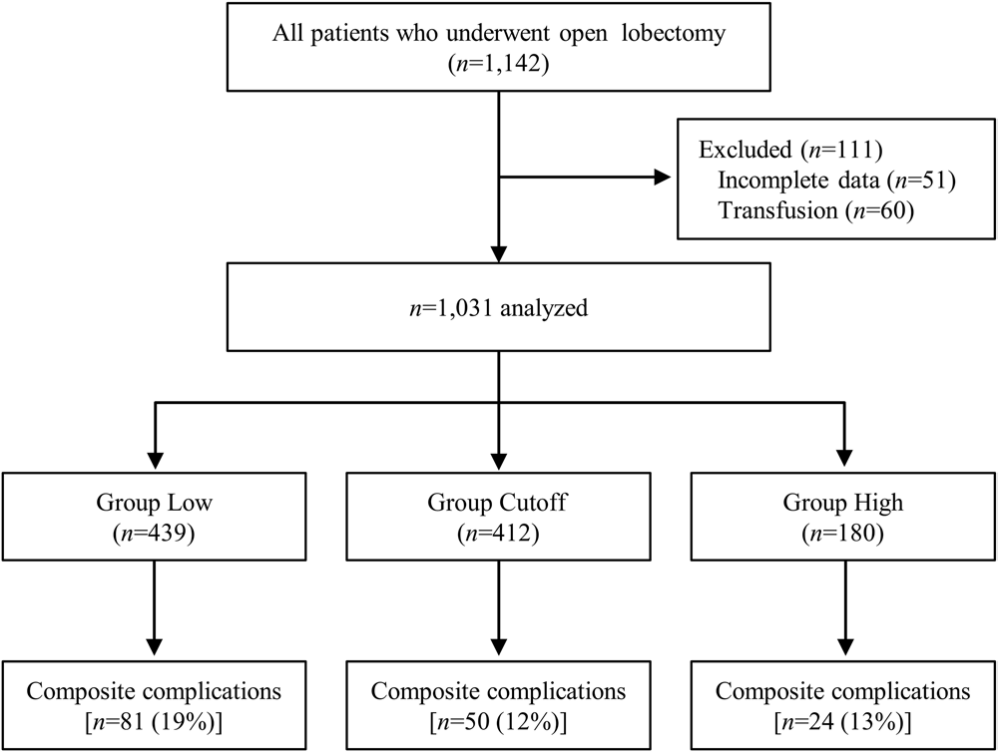
Flow diagram and the incidence of complications in each group.

This retrospective study was approved by the Institutional Review Board of Samsung Medical Centre, Chairperson Prof. Suk-Koo Lee, Seoul, Korea (IRB file number: SMC 2018-12-114-001, Date of approval January 09, 2019). Written consent was waived by the Institutional Review Board of Samsung Medical Centre for its retrospective nature. This manuscript adheres to the STROBE guidelines. We hypothesized that a certain range of infusion rate would decrease the composite complications within postoperative 30 days. Electronic medical records of patients (n = 1,142) who underwent open lung resection from January 2009 to December 2013 at our institution were reviewed by two reviewers. Conversion cases from VATS to open thoracotomy were included. Those who lacked blood loss and urine output data (n = 51) were excluded. Those who received intraoperative transfusion (n = 60) were also excluded because transfusion is a significant confounding factor of AKI^36^ and pulmonary complications^16^, and because our purpose was to determine optimal fluid infusion rate in regular lung resection surgery which usually does not accompany large blood loss and transfusion. For patients who had undergone more than one thoracic surgery during the study period, only data related to the first operation were included^29^. Finally 1,031 patients were analyzed. Patients were divided into Low (≤3 ml.kg^−1^.h^−1^), Cutoff (4–5 ml.kg^−1^.h^−1^), and High (≥6 ml.kg^−1^.h^−1^) intraoperative fluid groups (Fig. 2).

Other information was collected from the patients’ electronic medical records; For preoperative data, age, gender, comorbidities, American Society of Anesthesiologists (ASA) physical status, smoking status, alcohol consumption, clinical tumor, node, metastasis (TNM) stage, neoadjuvant chemoradiotherapy were collected^37^. Comorbid conditions included hypertension, diabetes mellitus, chronic renal disease (estimated glomerular filtration rate [eGFR] of <60 ml.min^−1^.1.73 m^2-1^), cerebrovascular disease, cardiac disease, and pulmonary dysfunction. Cerebrovascular diseases (history thereof) included cerebral infarction, cerebral hemorrhage, and dementia/Parkinson’s disease/Alzheimer’s disease^37^. Cardiac diseases included coronary artery disease and heart failure. Pulmonary dysfunction included lung disease (chronic obstructive pulmonary disease, bronchiectasis, asthma, interstitial lung disease) and preoperative forced expiratory volume in one second (FEV_1_) < 60% of predicted value. Current smokers were defined as patients who were still smoking or had stopped within 1 month before surgery^37^. Heavy drinking was defined as consuming 15 drinks or more per week for men and 8 drinks or more per week for women according to the Centers for Disease Control and Prevention (https://www.cdc.gov/alcohol/fact-sheets/alcohol-use.htm). For operative data, duration and type of surgery, intraoperative administration of an inotrope (dopamine or dobutamine continuous infusion) or vasopressor (phenylephrine or norepinephrine continuous infusion), method of postoperative analgesia were collected. For postoperative data, 24-hour postoperative input-output data, weight change after operation, postoperative complications up to discharge, in-hospital mortality, postoperative ICU stay, and days of hospitalization were collected^37^.

### Fluid management protocol

Patients were kept nil per os (NPO) from preoperative midnight. 5% DNK2 (*5*% dextrose with NaCL and KCL) was infused at 80 mL/h during NPO. The time frame of fluid monitoring was from the start of operation to postoperative 24 hours. The maintenance fluid used intraoperatively was Ringer’s lactate solution and was administered according to the attending anesthesiologist’s preference at a rate range of 1–11 ml.kg^−1^.h^−1^; 0.9% saline was not used. The intraoperative fluid amount was calculated as the total input minus the output. Input included crystalloid and colloid solutions. Output included estimated blood loss and urine output. Intraoperative fluid (input – output) was divided by the patient’s actual body weight and anesthesia duration to obtain the infusion rate.

Patients were divided into Low (≤3 ml.kg^−1^.h^−1^), Cutoff (4–5 ml.kg^−1^.h^−1^), and High (≥6 ml.kg^−1^.h^−1^) groups according to the intraoperative fluid amount. The cutoff values were determined by the minimum p-value method^38^; The cutoff which shows the lowest p-value with even distribution of patient population was 4–5 ml.kg^−1^.h^−1^. Therefore, the groups were divided according to the cutoff value of 4–5 ml.kg^−1^.h^−1^ (Cutoff group, χ^2^ = 7.129; degrees of freedom = 2).

In cases of intraoperative bleeding, 5% human albumin (Green Cross, Gyeonggi, Korea) or 6% hydroxyethyl starch in balanced solution (Volulyte, Fresenius Kabi, Seoul, Korea) was given to the patient. Transfusion was performed for effective resuscitation if the transfusion cutoff (hemoglobin <8 g/dl) was reached^37^. Postoperative fluid management was standardized at 1–2 ml.kg^−1^.h^−1^. Patients started sipping water several hours after the operation and they had porridge in the morning of the following day.

### Definition of postoperative complications

The primary endpoint was composite complications (pulmonary or renal complications). Pulmonary complications (up to postoperative 30 days) included acute respiratory distress syndrome (ARDS; based on the 2012 Berlin definition)^39^, pneumonia, and atelectasis requiring bronchoscopy. Pneumonia was defined by new pulmonary infiltrate with fever which was treated with intravenous antibiotics^40^. Atelectasis requiring bronchoscopy was determined by the surgical team^40^. Renal complications were defined using the Acute Kidney Injury Network (AKIN) classification. The time frame for recording changes from baseline was limited to 72 h postoperatively to ensure that the occurrence of AKI was related to the index procedure^29^. Regarding cases of myocardial infarction, only those requiring emergent revascularization were included. Regarding cerebral infarction, only symptomatic infarcts confirmed by radiologic study were included. Prolonged air leak and effusion was defined as >five days. Complications except AKI were recorded within postoperative 30 days.

### Anesthesia and postoperative management

Anesthesia and postoperative management were performed according to our institutional protocol. Most patients received balanced anesthesia, which was a combination of volatile anesthetic agents, non-depolarizing neuromuscular blocking agents, and a continuous intravenous infusion of remifentanil^37^. A protective ventilation protocol was performed for all patients (tidal volume 5–6 ml/kg of predicted body weight, plateau pressure <25 cmH_2_O, positive end-expiratory pressure 5–6 cmH_2_O). Inotrope (continuous infusion of dopamine or dobutamine) was administered when mean blood pressure is less than 65 mmHg with accompanying bradycardia. Vasopressor (continuous infusion of phenylephrine or norepinephrine) was administered when mean blood pressure is less than 65 mmHg without bradycardia. Postoperative analgesic methods were applied according to the surgeon’s preference and the existence of contraindications for regional analgesia^37^. Patients were encouraged to ambulate from postoperative day 1. They received a daily physiotherapy program, which included deep-breathing exercises, incentive spirometry, and chest physiotherapy, as applied by physiotherapists and attending nurses during ICU and ward stays^37^.

### Statistical analysis

The primary outcome was composite complications (pulmonary complications or AKI, chi-square test). We compared baseline, intraoperative, and postoperative variables among the Low, Cutoff, and High groups. Categorical variables were presented as number of patients (%) and compared using the chi-square test^29^. Continuous variables with normal distributions were presented as mean (SD), and variables with non-normal distributions were presented as median (interquartile range)^29^. Continuous variables were compared using either one-way analysis of variance (ANOVA) or the Kruskal-Wallis test as appropriate^29^. Multiple comparisons were adjusted by the Bonferroni correction. The Shapiro-Wilk test was used to analyze normally distributed data. Multivariable logistic regression analysis was performed to identify risk factors for postoperative complications^37^. Univariable analysis was performed for all variables and were further analyzed by multivariable analysis^37^. For all analyses, a two-sided P < 0.05 was considered significant. Data were analyzed using MedCalc for Windows (ver. 7.3; MedCalc Software, Mariakerke, Belgium) and SPSS software (ver. 25.0; IBM Corp., Chicago, IL, USA)^37^.

### Ethics approval and consent to participate

This retrospective study was approved by the Institutional Review Board of Samsung Medical Centre, Chairperson Prof. Suk-Koo Lee, Seoul, Korea (IRB file number: SMC 2018-12-114-001, Date of approval January 09, 2019). Written consent was waived for its retrospective nature.

## Data Availability

All data in this study are available from corresponding author on reasonable request.
